# Beyond BMI: central obesity identifies overlooked fatty liver disease risk in adults with normal body mass index undergoing routine health examinations

**DOI:** 10.3389/fpubh.2026.1852073

**Published:** 2026-06-25

**Authors:** Qingfeng He, Shang Li, Qian He, Yixiong Guo, Mei He, Suiyuan Li, Jiayi Luo, Sha Zhu

**Affiliations:** 1 Key Laboratory of Birth Defects and Related Diseases of Women and Children (Sichuan University), Ministry of Education, West China Second University Hospital, Sichuan University, Chengdu, China; 2 Health Examination Center, The Third People's Hospital of Chengdu, Chengdu, China; 3 Department of Gynecology and Obstetrics, West China Second University Hospital, Sichuan University, Chengdu, China; 4 Department of Pathology, West China Second University Hospital, Sichuan University, Chengdu, China; 5 Department of Outpatient, West China Second University Hospital, Sichuan University, Chengdu, China

**Keywords:** body mass index, central obesity, China, fatty liver disease, health examination, normal-weight obesity, opportunistic screening, waist circumference

## Abstract

**Background:**

Fatty liver disease (FLD) is increasingly common in the general population, yet risk stratification in routine health examinations still relies heavily on body mass index (BMI). Because BMI does not capture abdominal fat distribution, adults with normal BMI but excess central adiposity may be overlooked despite having elevated hepatic risk. We examined the association of combined BMI-waist phenotypes with ultrasound-diagnosed FLD in a Chinese health examination population.

**Methods:**

This retrospective cross-sectional study included 2,336 adults undergoing routine health examinations at the Health Examination Center of the Third People's Hospital of Chengdu, China. Participants were classified into four BMI-waist phenotypes: normal BMI/no central obesity, normal BMI/central obesity, overweight-obesity/no central obesity, and overweight-obesity/central obesity. The primary outcome was ultrasound-diagnosed FLD; moderate-to-severe steatosis was analyzed as a secondary outcome. Logistic regression models were fitted with sequential adjustment for major covariates. Additional analyses were conducted among adults with normal BMI, including a sensitivity analysis using waist-to-height ratio.

**Results:**

The overall prevalence of FLD was 28.3%. FLD prevalence increased across BMI-waist phenotypes, from 11.2% in the normal BMI/no central obesity group to 27.8% in the normal BMI/central obesity group, 27.9% in the overweight-obesity/no central obesity group, and 51.5% in the overweight-obesity/central obesity group. In the fully adjusted model, compared with the normal BMI/no central obesity group, the odds ratios (ORs) for FLD were 2.46 (95% CI: 1.73–3.48) for normal BMI/central obesity, 2.74 (95% CI: 2.01–3.72) for overweight-obesity/no central obesity, and 6.78 (95% CI: 5.26–8.79) for overweight-obesity/central obesity. Among adults with normal BMI, central obesity remained associated with FLD in the fully adjusted model (OR 2.48, 95% CI: 1.73–3.52). Similar patterns were observed for moderate-to-severe steatosis and in sensitivity analyses using waist-to-height ratio.

**Conclusion:**

Central obesity was associated with higher observed odds of ultrasound-diagnosed FLD beyond BMI alone. These findings apply to etiology-agnostic ultrasound-detected fatty liver rather than MASLD/MASH-specific disease and suggest that waist circumference may complement BMI in routine health examinations.

## 1 Introduction

Fatty liver disease (FLD), commonly identified as hepatic steatosis on imaging, has become one of the most common chronic liver disorders worldwide and a growing contributor to cardiometabolic disease burden (1, 2). Current estimates suggest that hepatic steatosis affects approximately one-third of adults globally, and its clinical relevance extends beyond liver-related outcomes because it is associated with type 2 diabetes, cardiovascular disease, chronic kidney disease, and increased healthcare use (3–6). Although FLD is often viewed as a complication of excess body weight, this view is incomplete. A substantial proportion of affected individuals are lean or nonobese by conventional body mass index (BMI) criteria, and this phenotype is particularly relevant in Asian populations, where metabolic risk may develop at lower BMI levels than in Western populations (7–12).

BMI remains widely used in clinical and public health screening because it is simple and reproducible, but it does not describe body-fat distribution. Individuals with normal BMI may still have excess abdominal or visceral adiposity, a pattern more closely linked to insulin resistance, cardiometabolic risk, and hepatic fat accumulation than overall body mass alone (13–17). Waist circumference is therefore increasingly regarded as a practical marker of central adiposity and may provide complementary information to BMI in routine risk assessment (18). Prior studies have shown that central obesity is associated with FLD after accounting for BMI, and that combining BMI with waist circumference may improve risk stratification compared with either measure alone (19, 20).

This issue has direct relevance for health examination practice in China. Large cross-sectional studies from Chinese health examination or general adult populations have reported a substantial burden of FLD and have repeatedly identified BMI, abdominal obesity, blood pressure, dysglycemia, and dyslipidemia as related factors (21–23). However, much of the existing evidence has focused on individual anthropometric indices or prediction markers, whereas fewer studies have examined simple joint BMI-waist phenotypes that can be immediately interpreted in routine screening (19, 24). In particular, it remains insufficiently clear whether waist-based assessment can identify adults with normal BMI who would otherwise be considered low risk, and whether this pattern extends beyond any steatosis to more clinically relevant steatosis severity. Because routine abdominal ultrasonography is pragmatic for health examinations but has limited sensitivity for mild steatosis, is operator-dependent, and is less accurate than MRI-based techniques, evidence from real-world ultrasound-based screening should be interpreted with appropriate methodological caution (25).

Therefore, the present study aimed to examine the association of combined BMI-waist phenotypes with ultrasound-diagnosed FLD among adults undergoing routine health examinations at a tertiary hospital health examination center in Chengdu, China. We further evaluated whether central obesity remained informative within the normal-BMI subgroup, assessed the association with moderate-to-severe steatosis, and performed a sensitivity analysis using waist-to-height ratio. By focusing on simple anthropometric phenotypes obtainable in routine practice, this study aimed to provide evidence relevant to upstream health-examination assessment of ultrasound-detected fatty liver.

## 2 Materials and methods

### 2.1 Study design and study population

This retrospective cross-sectional study used de-identified data from adults who underwent routine health examinations at the Health Examination Center of the Third People's Hospital of Chengdu between January 2023 and December 2025. The study was conducted and reported in accordance with the strengthening the reporting of observational studies in epidemiology (STROBE) recommendations. The health examination center serves adults undergoing self-referred or employer-organized routine examinations; therefore, the source population represents a single-center health examination cohort rather than a population-based sample. Participants were eligible if data were available on sex, age, anthropometric measurements, and abdominal ultrasonography. Individuals with missing data on the main exposure variables, outcome variables, or covariates required for the corresponding regression models were excluded from the relevant analyses. The final analytic sample was defined before model fitting.

Because this study was based on routinely collected health examination data, its purpose was to evaluate the association between anthropometric phenotypes and ultrasound-diagnosed FLD in real-world health examination setting rather than to establish causal relationships. All analyses were performed using anonymized records extracted from the electronic health examination database.

The study protocol was reviewed and approved by the Institutional Ethics Committee of the Third People's Hospital of Chengdu (Approval Number: cdth20230912). The study was conducted in accordance with the principles of the Declaration of Helsinki. As this was a retrospective study using de-identified data obtained from the electronic medical record and health examination system, the requirement for written informed consent was waived by the ethics committee.

### 2.2 Data collection and variable definitions

Baseline information was retrieved from the health examination database, including age, sex, smoking status, drinking status, physical activity, anthropometric measurements, blood pressure, laboratory parameters, medication use, medical history, and abdominal ultrasonography findings. Height and weight were used to calculate body mass index (BMI) as weight in kilograms divided by height in meters squared. Waist circumference was used as the primary indicator of abdominal adiposity because it provides clinically relevant information beyond BMI alone. Alcohol-related information was available only as broad self-reported drinking-status categories recorded in the health examination database, including no drinking, occasional drinking, and regular drinking.

For descriptive analyses, BMI was categorized according to Chinese adult criteria as underweight (< 18.5 kg/m^2^), normal weight (18.5– < 24.0 kg/ m^2^), overweight (24.0– < 28.0 kg/m^2^), or obesity (≥28.0 kg/m^2^) (26). For the main joint-phenotype analyses, BMI was further dichotomized as < 24.0 vs. ≥24.0 kg/m^2^ to distinguish normal-BMI from overweight-obesity status. Central obesity was defined using sex-specific waist circumference thresholds of ≥90 cm in men and ≥85 cm in women (27), consistent with commonly used cutoffs in Chinese adults. Overweight and obesity were combined for the primary BMI-waist phenotype definition to preserve a simple routine-assessment 2 × 2 BMI-waist framework and to maintain stable phenotype cell sizes after cross-classification with central obesity status; this combined category should be interpreted as excess BMI rather than as a homogeneous biological group. Based on these definitions, participants were classified into four BMI-waist phenotypes: normal BMI/no central obesity, normal BMI/central obesity, overweight-obesity/no central obesity, and overweight-obesity/central obesity.

A normal-BMI subgroup was defined as BMI < 24.0 kg/m^2^. In sensitivity analyses within this subgroup, central adiposity was alternatively defined using waist-to-height ratio (WHtR), calculated as waist circumference divided by height, with WHtR ≥0.50 used as the prespecified threshold (28), because this threshold is widely used as a simple public health boundary indicating that waist circumference should be less than one-half of height.

Hypertension and dysglycemia were defined for descriptive and subgroup analyses using composite clinical criteria. Hypertension was defined as systolic blood pressure ≥130 mmHg, diastolic blood pressure ≥85 mmHg, self-reported history of hypertension, or current use of antihypertensive medication. Dysglycemia was defined as fasting plasma glucose ≥5.6 mmol/L, hemoglobin A1c ≥5.7%, self-reported history of diabetes, or current use of glucose-lowering medication.

### 2.3 Outcome assessment

The primary outcome was ultrasound-diagnosed FLD. Abdominal ultrasonography was performed as part of the routine health examination using the health examination center's standard abdominal ultrasound systems, which were conventional color Doppler ultrasound diagnostic systems equipped with convex-array abdominal transducers (probe frequency: 2.0–5.0 MHz). Examinations were conducted by licensed ultrasound physicians or qualified sonographers following routine abdominal scanning protocols. Ultrasound reports were generated during the clinical examination workflow before construction of the research dataset. Abdominal ultrasonography findings recorded in the examination database were categorized as none, mild, or moderate-to-severe steatosis (25). The diagnosis and grading of hepatic steatosis were based on routine ultrasonographic features, including increased hepatic echogenicity relative to the renal cortex or spleen, posterior beam attenuation, and reduced visualization of intrahepatic vessels or the diaphragm. For the primary analysis, FLD was defined as the presence of any steatosis on ultrasonography (mild or moderate-to-severe) vs. no steatosis. For the secondary analysis, moderate-to-severe steatosis was analyzed as a separate binary outcome, with participants classified as moderate-to-severe vs. all others.

Abdominal ultrasonography was selected because it is a pragmatic and widely used method for steatosis detection in health examination and epidemiologic settings. However, ultrasonography is operator-dependent, has limited sensitivity for mild steatosis, and is less accurate than MRI-based techniques for quantifying liver fat. Because this was a retrospective analysis of routinely collected examination records, formal blinding of ultrasound operators to anthropometric data and formal interobserver reliability testing could not be verified from the available database.

### 2.4 Statistical analysis

Continuous variables were summarized as mean ± standard deviation, and categorical variables as number (percentage). Baseline characteristics were compared between participants with and without FLD using Welch's two-sample *t*-test for continuous variables and Pearson's chi-squared test for categorical variables. Baseline comparisons were interpreted descriptively, with attention to the magnitude and clinical relevance of group differences rather than statistical significance alone.

The association between BMI-waist phenotypes and FLD was examined using binary logistic regression. In the main analysis, the reference group was normal BMI/no central obesity. Three models were fitted: model 1 was unadjusted; Model 2 was adjusted for age and sex; and Model 3 was additionally adjusted for smoking status, drinking status, and physical activity. The same modeling strategy was used for the secondary outcome of moderate-to-severe steatosis. To address potential heterogeneity within the combined overweight-obesity category, we conducted an additional supplementary analysis in which BMI was further categorized as normal BMI, overweight, and obesity according to Chinese adult criteria. These BMI categories were cross-classified with central obesity status to generate an expanded BMI-waist phenotype. The same logistic regression framework was applied to ultrasound-diagnosed FLD and moderate-to-severe steatosis, using normal BMI without central obesity as the reference group.

Given that alcohol use was recorded only in broad categories, we conducted an additional sensitivity analysis excluding participants who reported regular drinking. The main BMI-waist phenotype models were refitted for ultrasound-diagnosed FLD and moderate-to-severe steatosis in this restricted sample, using the same modeling sequence except that drinking status was not included in Model 3. To evaluate whether the associations were materially changed after accounting for broader metabolic status, we performed another supplementary sensitivity analysis by further adjusting Model 3 for hypertension and dysglycemia. Because these variables may partly reflect metabolic consequences of adiposity.

To assess whether central obesity remained associated with FLD among individuals without excess BMI, a normal-BMI subgroup analysis was performed using the same three-model framework, with no central obesity as the reference category. In this subgroup, a sensitivity analysis replaced waist circumference-defined central obesity with WHtR-defined central adiposity (WHtR ≥0.50 vs. < 0.50) in an otherwise similarly adjusted model. Odds ratios (ORs) and 95% confidence intervals (CIs) were reported.

To visualize the graded association between waist circumference and ultrasound-diagnosed FLD within the normal-BMI subgroup, waist circumference was modeled using natural cubic splines in a multivariable logistic regression model. The spline specification used three degrees of freedom, corresponding to two internal knots placed at the 33.3rd and 66.7th percentiles of waist circumference within the normal-BMI subgroup. The model was adjusted for sex, age, smoking status, drinking status, and physical activity. Evidence for nonlinearity was evaluated using a likelihood ratio test comparing the spline model with a corresponding model containing waist circumference as a single linear term. Adjusted fitted probabilities and 95% CIs were derived using marginal standardization over the observed distribution of adjustment covariates. For visualization, predictions were restricted to the 2.5th−97.5th percentile range of waist circumference to reduce instability at the distributional extremes. Model convergence and the distribution of fitted probabilities were checked.

Because this was a cross-sectional study, all odds ratios were interpreted as measures of association rather than prediction or future risk. In addition to odds ratios, absolute prevalence estimates across BMI-waist phenotypes were calculated to aid clinical interpretation. The proportion of ultrasound-diagnosed FLD cases occurring among participants with normal BMI was also calculated to describe the extent to which FLD cases would not be captured by a BMI ≥24.0 kg/m^2^ criterion alone.

Potential collinearity between BMI and waist circumference was assessed using Pearson and Spearman correlation coefficients and classical variance inflation factors. Variance inflation factors were calculated by regressing each anthropometric variable on the other anthropometric variable and the main covariates, including age, sex, smoking status, drinking status, and physical activity. These diagnostics were used to assess the stability of models involving related anthropometric constructs.

Subgroup analyses within the normal-BMI population were further performed according to sex, age group (< 45 vs. ≥45 years), hypertension, and dysglycemia. Within each subgroup, the association between central obesity and FLD was estimated using multivariable logistic regression adjusted for the remaining covariates, except for the stratifying variable itself. *P*-values for interaction were calculated using likelihood ratio tests comparing models with and without the corresponding interaction term. All statistical tests were two-sided, and a *P*-value < 0.05 was considered statistically significant. Analyses were conducted using R version 4.5.3 (R foundation for statistical computing, Vienna, Austria).

## 3 Results

### 3.1 Participant characteristics

A total of 2,336 adults were included in the analysis, of whom 660 had ultrasound-diagnosed FLD, corresponding to an overall prevalence of 28.3% (Table 1).

**Table 1:** Baseline characteristics according to ultrasound-diagnosed fatty liver disease.

Characteristic	No *N* = 1,676	Yes *N* = 660	*P*-value
Age, years	44.56 ± 11.85	49.45 ± 11.59	< 0.001
Sex			
Female	871 (52%)	168 (25%)	< 0.001
Male	805 (48%)	492 (75%)	
Smoking status			
Current	231 (14%)	136 (21%)	< 0.001
Former	272 (16%)	147 (22%)	
Never	1,173 (70%)	377 (57%)	
Drinking status			
No	939 (56%)	313 (47%)	< 0.001
Occasional	523 (31%)	257 (39%)	
Regular	214 (13%)	90 (14%)	
Physical activity			
High	354 (21%)	125 (19%)	0.483
Low	426 (25%)	169 (26%)	
Moderate	896 (53%)	366 (55%)	
Body mass index, kg/m^2^	23.12 ± 2.88	25.79 ± 2.90	< 0.001
BMI category (Chinese criteria)			
Underweight	91 (5.4%)	7 (1.1%)	< 0.001
Normal weight	951 (57%)	173 (26%)	
Overweight	551 (33%)	327 (50%)	
Obesity	83 (5.0%)	153 (23%)	
Waist circumference, cm	82.36 ± 10.06	92.73 ± 9.89	< 0.001
Central obesity	537 (32%)	442 (67%)	< 0.001
Systolic blood pressure, mmHg	120.03 ± 12.91	128.14 ± 12.72	< 0.001
Diastolic blood pressure, mmHg	70.75 ± 8.45	75.53 ± 8.15	< 0.001
Fasting plasma glucose, mmol/L	4.91 ± 0.53	5.36 ± 0.55	< 0.001
Hemoglobin A1c, %	5.12 ± 0.30	5.32 ± 0.31	< 0.001
Total cholesterol, mmol/L	4.93 ± 0.70	5.33 ± 0.78	< 0.001
Triglycerides, mmol/L	1.45 ± 0.59	2.05 ± 0.85	< 0.001
High-density lipoprotein cholesterol, mmol/L	1.38 ± 0.20	1.25 ± 0.19	< 0.001
Low-density lipoprotein cholesterol, mmol/L	2.88 ± 0.58	3.11 ± 0.61	< 0.001
Alanine aminotransferase, U/L	22.58 ± 9.14	39.54 ± 10.85	< 0.001
Aspartate aminotransferase, U/L	19.92 ± 5.21	26.28 ± 5.82	< 0.001
Gamma-glutamyl transferase, U/L	26.30 ± 11.56	37.52 ± 10.73	< 0.001
Platelet count, × 10^9^/L	233.60 ± 41.95	234.28 ± 43.57	0.733
Serum uric acid, μmol/L	323.36 ± 54.59	367.43 ± 52.43	< 0.001
Hypertension	493 (29%)	349 (53%)	< 0.001
Dysglycemia	220 (13%)	262 (40%)	< 0.001

Continuous variables are presented as mean ± standard deviation, and categorical variables as n (%). Fatty liver disease was defined according to abdominal ultrasonography findings. Central obesity was defined as waist circumference ≥90 cm in men and ≥85 cm in women. Hypertension was defined as systolic blood pressure ≥130 mmHg, diastolic blood pressure ≥85 mmHg, self-reported history of hypertension, or current use of antihypertensive medication. Dysglycemia was defined as fasting plasma glucose ≥5.6 mmol/L, hemoglobin A1c ≥5.7%, self-reported history of diabetes, or current use of glucose-lowering medication. P-values were derived from Welch's two-sample t-tests for continuous variables and Pearson's chi-squared tests for categorical variables. BMI, body mass index; mmHg, millimeters of mercury; FPG, fasting plasma glucose; HbA1c, hemoglobin A1c; HDL-C, high-density lipoprotein cholesterol; LDL-C, low-density lipoprotein cholesterol; ALT, alanine aminotransferase; AST, aspartate aminotransferase; GGT, gamma-glutamyl transferase.

Compared with participants without ultrasound-diagnosed FLD, those with FLD were older (49.45 ± 11.59 vs. 44.56 ± 11.85 years), were more often male (75 vs. 48%), and showed different distributions of smoking and drinking status (Table 1). Participants with FLD also had higher body mass index (BMI; 25.79 ± 2.90 vs. 23.12 ± 2.88 kg/m^2^), larger waist circumference (92.73 ± 9.89 vs. 82.36 ± 10.06 cm), and a higher prevalence of central obesity (67 vs. 32%). In addition, they had higher systolic and diastolic blood pressure, fasting plasma glucose, hemoglobin A1c, total cholesterol, triglycerides, low-density lipoprotein cholesterol, alanine aminotransferase, aspartate aminotransferase, gamma-glutamyl transferase, and serum uric acid, and lower high-density lipoprotein cholesterol. Hypertension and dysglycemia were also more common in the FLD group (53 vs. 29% and 40 vs. 13%, respectively). Physical activity and platelet count did not differ materially between groups (*P* = 0.483 and *P* = 0.733, respectively).

### 3.2 Prevalence of ultrasound-diagnosed FLD across BMI-waist phenotypes

When participants were classified into four BMI-waist phenotypes, 959 were categorized as normal BMI/no central obesity, 263 as normal BMI/central obesity, 398 as overweight-obesity/no central obesity, and 716 as overweight-obesity/central obesity (Supplementary Table S1).

**Figure 1:**
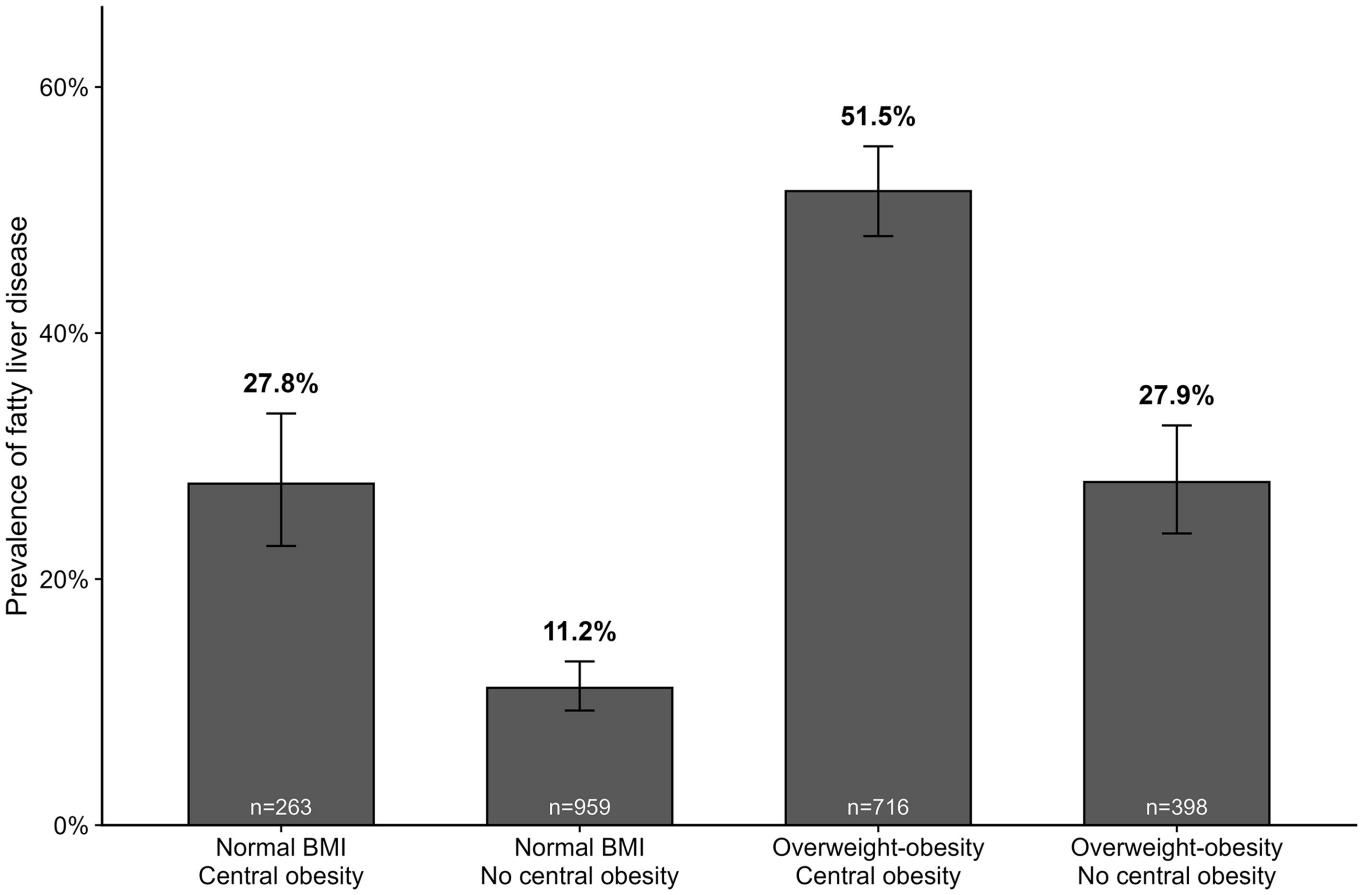
Prevalence of ultrasound-diagnosed fatty liver disease across BMI-waist phenotypes. Bars show the absolute prevalence of ultrasound-diagnosed fatty liver disease across the four BMI-waist phenotype groups, and error bars indicate 95% confidence intervals. The figure shows a graded prevalence pattern, with the lowest prevalence in participants with normal BMI/no central obesity, similar prevalence in participants with normal BMI/central obesity and overweight-obesity/no central obesity, and the highest prevalence in participants with both overweight-obesity and central obesity. BMI categories were defined according to Chinese adult criteria. Central obesity was defined as waist circumference ≥90 cm in men and ≥85 cm in women.

The absolute prevalence of ultrasound-diagnosed FLD differed substantially across BMI-waist phenotypes. FLD was present in 107 of 959 participants (11.2%) in the normal BMI/no central obesity group, 73 of 263 participants (27.8%) in the normal BMI/central obesity group, 111 of 398 participants (27.9%) in the overweight-obesity/no central obesity group, and 369 of 716 participants (51.5%) in the overweight-obesity/central obesity group (Figure 1). Thus, the absolute prevalence among participants with normal BMI and central obesity was 16.6 percentage points higher than that among participants with normal BMI and no central obesity, and was similar to the prevalence observed among participants with overweight-obesity but no central obesity.

A similar graded pattern was observed for ultrasound steatosis severity. The proportion of moderate-to-severe steatosis was 1.0% in the normal BMI/no central obesity group, 4.9% in the normal BMI/central obesity group, 7.0% in the overweight-obesity/no central obesity group, and 20.0% in the overweight-obesity/central obesity group (Supplementary Table S1; Figure S1). Figure S1 also shows that the distribution of steatosis grades shifted toward more severe categories across the BMI-waist phenotype groups.

### 3.3 Association of BMI-waist phenotypes with ultrasound-diagnosed FLD

In logistic regression analyses, BMI-waist phenotypes were associated with ultrasound-diagnosed FLD (Table 2). Using the normal BMI/no central obesity group as the reference, the fully adjusted ORs were 2.46 (95% CI: 1.73–3.48) for normal BMI/central obesity, 2.74 (95% CI: 2.01–3.72) for overweight-obesity/no central obesity, and 6.78 (95% CI: 5.26–8.79) for overweight-obesity/central obesity.

**Table 2:** Association of BMI-waist phenotypes with fatty liver disease, including the normal-BMI subgroup analysis.

Variable	Model 1	Model 2	Model 3
Panel A. main analysis			
Normal BMI/central obesity	3.06 (2.18, 4.28)	2.42 (1.71, 3.42)	2.46 (1.73, 3.48)
Overweight-obesity/no central obesity	3.08 (2.29, 4.15)	2.71 (2.00, 3.68)	2.74 (2.01, 3.72)
Overweight-obesity/central obesity	8.47 (6.62, 10.90)	6.74 (5.23, 8.74)	6.78 (5.26, 8.79)
Panel B. Normal-BMI subgroup analysis			
Central obesity (vs. no central obesity)	3.06 (2.18, 4.28)	2.40 (1.68, 3.39)	2.48 (1.73, 3.52)

Values are odds ratios (95% confidence intervals) estimated using binary logistic regression. In Panel A, “Normal BMI / No central obesity” was used as the reference group. In Panel B, analyses were restricted to participants with BMI < 24 kg/m^2^ according to Chinese adult criteria, and “No central obesity” was used as the reference group. Model 1 was unadjusted. Model 2 was adjusted for age and sex. Model 3 was additionally adjusted for smoking status, drinking status, and physical activity. Fatty liver disease was defined according to abdominal ultrasonography findings. Central obesity was defined as waist circumference ≥90 cm in men and ≥85 cm in women. BMI, body mass index; OR, odds ratio; CI, confidence interval.

The association estimates were similar in direction across the less adjusted models. In Model 1, the corresponding ORs were 3.06 (95% CI: 2.18–4.28), 3.08 (95% CI: 2.29–4.15), and 8.47 (95% CI: 6.62–10.90). In Model 2, they were 2.42 (95% CI: 1.71–3.42), 2.71 (95% CI: 2.00–3.68), and 6.74 (95% CI: 5.23–8.74), respectively.

### 3.4 Central obesity and FLD among participants with normal BMI

Among participants with normal BMI, central obesity was associated with higher odds of ultrasound-diagnosed FLD (Table 2, Panel B). The ORs for central obesity vs. no central obesity were 3.06 (95% CI: 2.18–4.28) in Model 1, 2.40 (95% CI: 1.68–3.39) in Model 2, and 2.48 (95% CI: 1.73–3.52) in Model 3.

**Figure 2:**
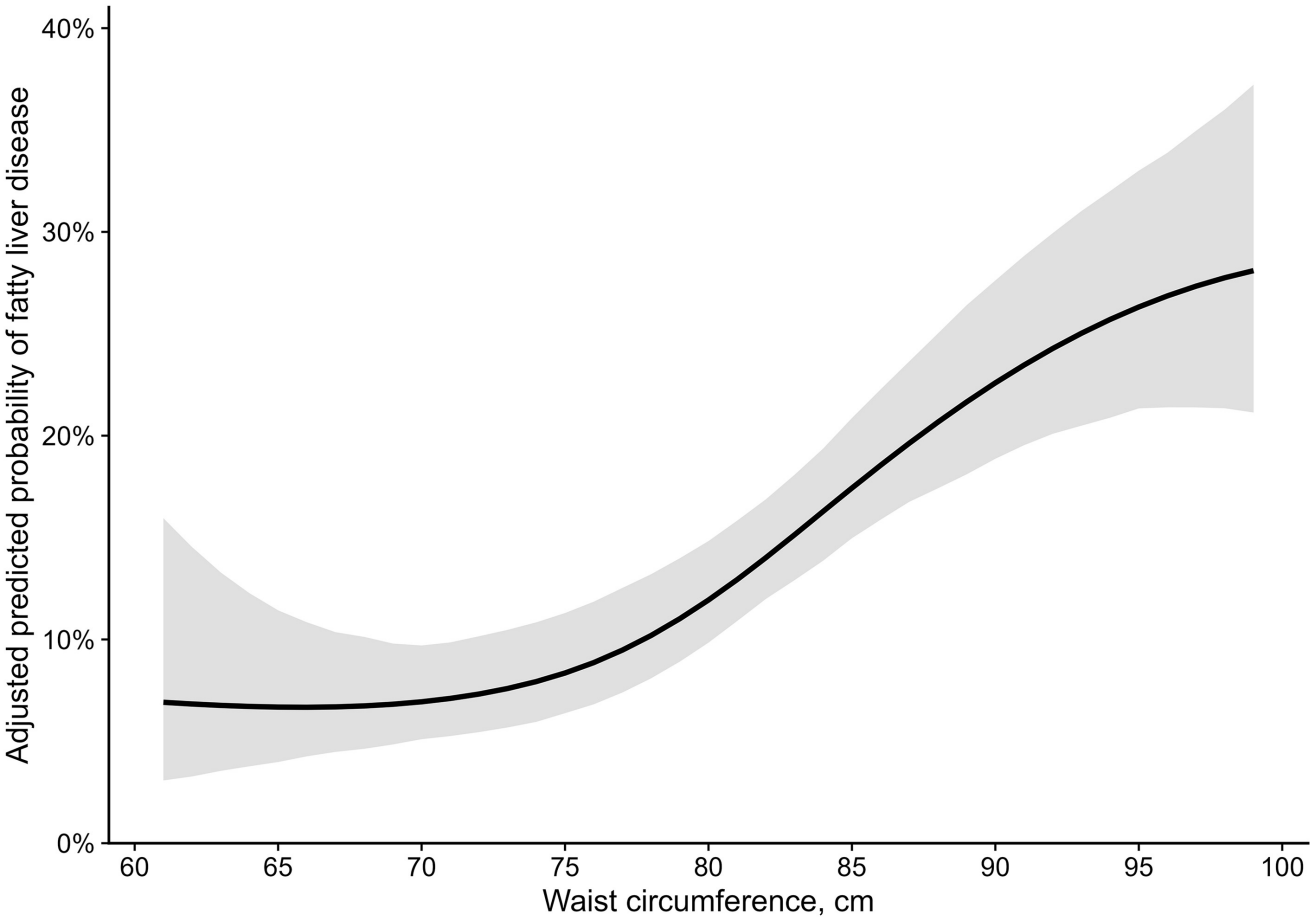
Adjusted fitted probability of ultrasound-diagnosed fatty liver disease according to waist circumference among adults with normal BMI. The solid curve shows the adjusted fitted probability of ultrasound-diagnosed fatty liver disease across waist circumference values among participants with normal BMI. The shaded area indicates the 95% confidence interval. Estimates were derived from a multivariable logistic regression model using natural cubic splines for waist circumference with three degrees of freedom, corresponding to two internal knots placed at the 33.3rd and 66.7th percentiles of waist circumference. The model was adjusted for sex, age, smoking status, drinking status, and physical activity. Predictions were displayed across the 2.5th to 97.5th percentile range of waist circumference to reduce instability at the distributional extremes. The likelihood ratio test did not show evidence of nonlinearity (*P* for nonlinearity = 0.220).

Consistent with the categorical analysis, the adjusted spline curve showed a graded increase in the fitted probability of ultrasound-diagnosed FLD with higher waist circumference among participants with normal BMI (Figure 2). The likelihood ratio test did not show evidence of departure from linearity (*P* for nonlinearity = 0.220), suggesting that the association was approximately monotonic over the plotted waist-circumference range. This visual pattern supported the observation that abdominal adiposity provided additional cross-sectional information within the lower-BMI range.

### 3.5 Association with moderate-to-severe steatosis

The same phenotype pattern was observed when moderate-to-severe steatosis was used as the outcome (Table 3). In the fully adjusted model, compared with the normal BMI/no central obesity group, the ORs were 3.73 (95% CI: 1.61–8.90) for normal BMI/central obesity, 6.15 (95% CI: 3.04–13.48) for overweight-obesity/no central obesity, and 18.13 (95% CI: 9.91–37.22) for overweight-obesity/central obesity.

**Table 3:** Association of BMI-waist phenotypes with moderate-to-severe steatosis.

Variable	Model 1	Model 2	Model 3
Normal BMI/central obesity	4.93 (2.15, 11.69)	3.71 (1.60, 8.85)	3.73 (1.61, 8.90)
Overweight-obesity/no central obesity	7.18 (3.57, 15.68)	6.10 (3.02, 13.38)	6.15 (3.04, 13.48)
Overweight-obesity/central obesity	24.31 (13.38, 49.67)	18.01 (9.85, 36.95)	18.13 (9.91, 37.22)

Values are odds ratios (95% confidence intervals) estimated using binary logistic regression, with “Normal BMI / No central obesity” as the reference group. The outcome was moderate-to-severe steatosis on abdominal ultrasonography. Model 1 was unadjusted. Model 2 was adjusted for age and sex. Model 3 was additionally adjusted for smoking status, drinking status, and physical activity. BMI, body mass index; OR, odds ratio; CI, confidence interval.

The corresponding estimates in Model 1 were 4.93 (95% CI: 2.15–11.69), 7.18 (95% CI: 3.57–15.68), and 24.31 (95% CI: 13.38–49.67). In Model 2, they were 3.71 (95% CI: 1.60–8.85), 6.10 (95% CI: 3.02–13.38), and 18.01 (95% CI: 9.85–36.95), respectively.

### 3.6 Sensitivity and subgroup analyses

In the sensitivity analysis restricted to participants with normal BMI, redefining central adiposity using a waist-to-height ratio of at least 0.50 yielded a similar association with ultrasound-diagnosed FLD, with an adjusted OR of 2.65 (95% CI: 1.88–3.78) (Supplementary Table S2).

After excluding participants who reported regular drinking, 2,032 participants remained, including 570 with ultrasound-diagnosed FLD and 170 with moderate-to-severe steatosis. The associations were directionally consistent with the primary analysis (Supplementary Table S4). In Model 3, the ORs for ultrasound-diagnosed FLD were 2.23 (95% CI: 1.52–3.26) for normal BMI/central obesity, 2.51 (95% CI: 1.80–3.50) for overweight-obesity/no central obesity, and 7.04 (95% CI: 5.37–9.29) for overweight-obesity/central obesity. In a further sensitivity analysis additionally adjusting for hypertension and dysglycemia, the associations were attenuated but remained directionally consistent (Supplementary Table S5). For ultrasound-diagnosed FLD, the ORs were 2.36 (95% CI: 1.66–3.35) for normal BMI/central obesity, 2.42 (95% CI: 1.77–3.31) for overweight-obesity/no central obesity, and 5.39 (95% CI: 4.12–7.10) for overweight-obesity/central obesity. Similar patterns were observed for moderate-to-severe steatosis.

**Figure 3:**
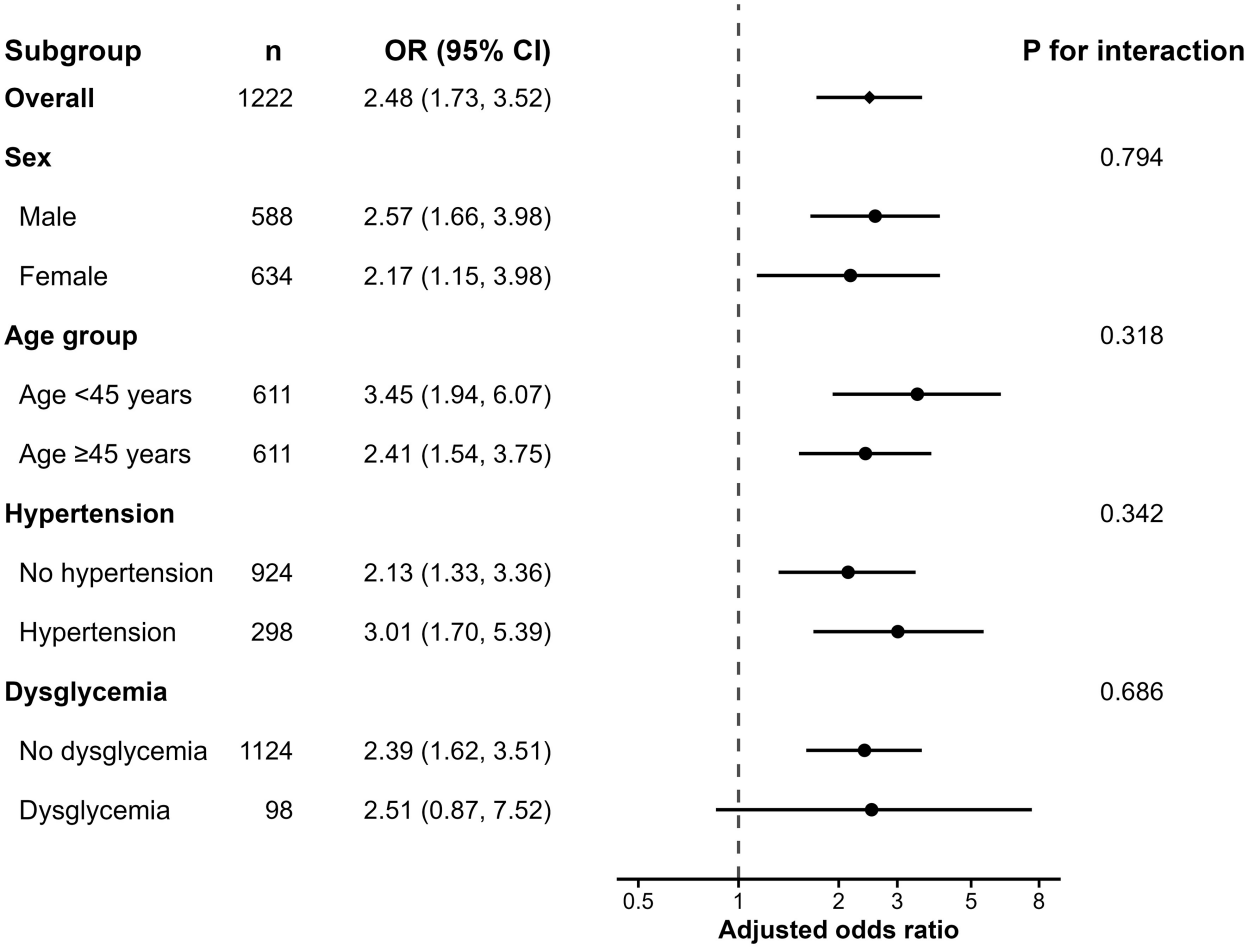
Subgroup analysis of the association between central obesity and ultrasound-diagnosed fatty liver disease among adults with normal BMI. Points represent adjusted odds ratios, and horizontal lines indicate 95% confidence intervals for the association between central obesity and ultrasound-diagnosed fatty liver disease within each subgroup. Models were adjusted for age, sex, smoking status, drinking status, and physical activity, except for the corresponding stratifying variable. *P*-values for interaction were calculated using likelihood ratio tests comparing models with and without the interaction term. The figure shows directionally consistent associations across subgroups defined by sex, age group, hypertension, and dysglycemia, with no statistically significant interaction observed.

In subgroup analyses among participants with normal BMI, the association between central obesity and ultrasound-diagnosed FLD was directionally consistent across strata defined by sex, age group, hypertension, and dysglycemia (Figure 3). The adjusted OR was 2.57 (95% CI: 1.66–3.98) in men and 2.17 (95% CI: 1.15–3.98) in women. The corresponding estimates were 3.45 (95% CI: 1.94–6.07) among participants aged < 45 years and 2.41 (95% CI: 1.54–3.75) among those aged ≥45 years; 2.13 (95% CI: 1.33–3.36) among those without hypertension and 3.01 (95% CI: 1.70–5.39) among those with hypertension; and 2.39 (95% CI: 1.62–3.51) among those without dysglycemia and 2.51 (95% CI: 0.87–7.52) among those with dysglycemia. No interaction reached statistical significance across these subgroup variables.

### 3.7 Practical interpretation of FLD case distribution by BMI status

Among the 660 participants with ultrasound-diagnosed FLD, 180 had normal BMI, accounting for 27.3% of all FLD cases. Within this lower-BMI FLD group, 73 participants had central obesity, corresponding to 40.6% of FLD cases among participants with normal BMI and 11.1% of all FLD cases. These descriptive findings indicate that a BMI ≥24 kg/m^2^ criterion alone would not capture a meaningful subset of participants with ultrasound-diagnosed FLD in this health examination cohort.

### 3.8 Additional analysis separating overweight and obesity

In the additional supplementary analysis separating overweight and obesity, the findings were consistent with the primary BMI-waist phenotype analysis (Supplementary Table S3). In the fully adjusted model, participants with normal BMI and central obesity had higher odds of ultrasound-diagnosed FLD (OR 2.46, 95% CI: 1.73–3.48), with an estimate comparable to that of overweight participants without central obesity (OR 2.40, 95% CI: 1.74–3.30). The corresponding ORs were 5.45 (95% CI: 4.15–7.19) for overweight participants with central obesity, 10.16 (95% CI: 4.67–22.82) for participants with obesity but no central obesity, and 12.00 (95% CI: 8.37–17.36) for participants with both obesity and central obesity.

Similar patterns were observed for moderate-to-severe steatosis. In the fully adjusted model, the ORs were 3.74 (95% CI: 1.61–8.92) for normal BMI/central obesity, 4.95 (95% CI: 2.35–11.13) for overweight/no central obesity, 12.36 (95% CI: 6.59–25.82) for overweight/central obesity, 22.76 (95% CI: 7.50–66.45) for obesity/no central obesity, and 36.96 (95% CI: 19.25–78.50) for obesity/central obesity (Supplementary Table S3).

### 3.9 Collinearity diagnostics

In collinearity diagnostics, BMI and waist circumference were moderately correlated (Pearson *r* = 0.616; Spearman ρ = 0.602; both *P* < 0.001). Classical variance inflation factors were 1.63 for BMI and 1.95 for waist circumference, suggesting no evidence of problematic multicollinearity between these anthropometric variables.

## 4 Discussion

In this cross-sectional study of adults undergoing routine health examinations, BMI-waist phenotypes were associated with the observed prevalence and odds of ultrasound-diagnosed FLD. The absolute prevalence of FLD was lowest in participants with normal BMI and no central obesity, higher and nearly identical in participants with normal BMI/central obesity and overweight-obesity/no central obesity, and highest in those with both overweight-obesity and central obesity. Similar patterns were observed in the normal-BMI subgroup, in the waist-to-height ratio sensitivity analysis, after excluding participants who reported regular drinking, and for moderate-to-severe steatosis. These findings suggest that waist circumference may complement BMI when evaluating adults with possible hepatic steatosis in routine health examination settings; however, they should not be interpreted as evidence of causality, disease progression, or validated screening performance. It should also be noted that the outcome in this study was etiology-agnostic ultrasound-detected fatty liver rather than strictly defined MASLD or MASH, because the information required for etiologic classification was not fully available in the health examination database.

This distinction is important when placing the results in the context of the current steatotic liver disease nomenclature and MASLD/MASH framework (29). Although this study does not strictly define MASLD or MASH, it may inform upstream health-examination assessment of ultrasound-detected fatty liver within the broader landscape of steatotic liver disease. Existing studies have shown that hepatic steatosis is common worldwide and that a meaningful proportion of affected individuals do not meet conventional BMI thresholds for obesity (1, 4, 6, 8, 30–33). In Asian populations, metabolic dysfunction and visceral adiposity may occur at lower BMI levels than in Western populations (10, 11, 34). Our study adds evidence from a Chinese health examination cohort by focusing on a simple joint BMI-waist phenotype that is directly interpretable in routine practice.

The finding with the greatest practical relevance was the similarity between the normal BMI/central obesity phenotype and the overweight-obesity/no central obesity phenotype. In absolute terms, FLD prevalence among participants with normal BMI and central obesity was 16.6 percentage points higher than that among participants with normal BMI and no central obesity, and was almost identical to the prevalence observed among participants with overweight-obesity but no central obesity. In addition, 27.3% of all FLD cases occurred among participants with normal BMI, and 40.6% of these lower-BMI FLD cases had central obesity. These descriptive results do not establish the diagnostic performance of waist circumference, but they indicate that a BMI-only approach may leave a subset of adults with ultrasound-diagnosed FLD less apparent in health examination settings.

Several biological considerations may help explain why waist circumference provided information beyond BMI. BMI reflects overall body size and cannot distinguish fat mass from lean mass or visceral from subcutaneous adiposity (18, 35). Waist circumference more closely reflects abdominal fat accumulation, which is linked to insulin resistance, free fatty acid flux to the liver, low-grade inflammation, and ectopic fat deposition (14, 15, 36, 37). These pathways may be relevant even when overall body weight remains below the conventional overweight threshold. At the same time, waist circumference should not be viewed as independent of broader metabolic dysfunction. Residual metabolic confounding remains possible because direct measures of insulin resistance, detailed diet, sedentary behavior, socioeconomic status, lipid-lowering therapy, and more detailed alcohol exposure were not available.

The sex distribution of FLD in this cohort also deserves cautious interpretation. Men comprised a larger proportion of participants with ultrasound-diagnosed FLD than women. Although sex-specific waist circumference thresholds were used and subgroup analyses showed directionally consistent associations in men and women, sex-related differences in fat distribution, hormonal status, and metabolic risk may still influence the observed associations (38, 39). Menopausal status was not available in the database, which may be relevant for interpreting adiposity and metabolic risk among women (40). Therefore, the sex-stratified findings should be considered supportive rather than definitive.

The results for moderate-to-severe steatosis were directionally consistent with the primary outcome, but they require particular caution. The odds ratios were larger than those for any ultrasound-diagnosed FLD, especially among participants with both overweight-obesity and central obesity. However, moderate-to-severe steatosis involved fewer events than the primary outcome, and some estimates had wide confidence intervals. These findings may therefore be affected by sparse data, reduced precision, and model instability. They should be interpreted as supportive evidence that the BMI-waist phenotype pattern extends to more severe ultrasound categories, rather than as precise estimates of effect magnitude.

The spline and sensitivity analyses provide additional internal consistency but should also be interpreted conservatively. In the normal-BMI subgroup, the fitted probability of ultrasound-diagnosed FLD increased with greater waist circumference, although there was no statistical evidence of departure from linearity. The waist-to-height ratio sensitivity analysis yielded a similar association, suggesting that the main finding was not dependent on a single waist-based definition. These analyses support a graded cross-sectional association between abdominal adiposity and FLD within the lower-BMI range, but they cannot establish temporality or future risk.

This study has several strengths, including a real-world health examination setting, a moderately sized sample, simple anthropometric phenotypes, and consistency across primary, subgroup, sensitivity, and supplementary analyses. The additional analysis separating overweight and obesity also reduced concern that the primary overweight-obesity category masked substantial heterogeneity. Nevertheless, the limitations are important. First, the cross-sectional design precludes causal inference, does not establish temporality, and cannot exclude reverse causation. In addition, because ultrasound-diagnosed FLD was relatively common in this cohort, odds ratios may overstate relative differences compared with prevalence ratios or risk ratios; therefore, the ORs should be interpreted as measures of cross-sectional association rather than relative risks. Second, the sample was drawn from a single tertiary-hospital health examination center. Participants undergoing self-referred or employer-organized examinations may differ from the general population in health awareness, socioeconomic status, healthcare access, and metabolic disease burden, raising the possibility of healthy screen bias and limiting external validity. Generalization beyond this setting also requires caution because BMI distributions, waist circumference thresholds, alcohol-use patterns, cardiometabolic profiles, healthcare access, and background liver disease etiologies may vary across regions of China, ethnic groups, and healthcare systems. Therefore, the findings are most directly applicable to adults undergoing routine health examinations in similar Chinese urban hospital-based settings and should be extrapolated cautiously to community-based populations, other regions of China, and non-Asian populations. Third, outcome classification relied entirely on abdominal ultrasonography. Ultrasonography is pragmatic and widely used in health examination settings, but it is operator-dependent, has limited sensitivity for mild steatosis, may be less sensitive in individuals with obesity, and is less accurate than magnetic resonance-based techniques for quantifying liver fat (25). It also cannot reliably distinguish steatohepatitis from simple steatosis, stage fibrosis, or provide histopathologic confirmation. Misclassification of mild steatosis and inability to characterize fibrosis may have influenced the observed associations. Fourth, the available data did not allow strict etiologic classification within the current steatotic liver disease framework. Alcohol use was recorded only as broad drinking-status categories, without quantitative intake, drinking duration, binge-drinking information, validated alcohol-use assessment, or formal evaluation for alcohol-related liver disease. Although drinking status was adjusted for in the primary model and a sensitivity analysis excluding regular drinkers showed directionally consistent results, alcohol-related or mixed-etiology fatty liver could not be fully excluded. Residual confounding from alcohol consumption may remain because the available drinking-status categories did not capture drinking quantity, duration, pattern, or lifetime exposure. Therefore, the findings should be interpreted as applying to etiology-agnostic ultrasound-detected fatty liver rather than MASLD/MASH-specific disease. Fifth, residual confounding remains possible. The additional sensitivity analysis adjusting for hypertension and dysglycemia showed directionally consistent associations, but these variables are only partial markers of broader metabolic status and may also lie on the pathway between adiposity and hepatic steatosis. Important factors such as insulin resistance, dietary intake, sedentary behavior, medication use, socioeconomic status, menopausal status, and other metabolic or lifestyle variables were not fully captured. Thus, the study cannot determine whether waist circumference contributes independently beyond the broader metabolic milieu.

In conclusion, among adults undergoing routine health examinations, BMI-waist phenotypes were associated with the observed prevalence and odds of ultrasound-diagnosed FLD. Adults with normal BMI but central obesity had a higher observed burden of FLD than those with normal BMI and no central obesity, and their estimates were similar to those of overweight-obese adults without central obesity. Waist circumference may therefore complement BMI when evaluating adults with possible hepatic steatosis in health examination settings. Further population-based and longitudinal studies using more precise liver fat and fibrosis assessment are needed before these findings can be translated into formal screening or clinical implementation strategies.

## Data Availability

The raw data supporting the conclusions of this article will be made available by the authors, without undue reservation.
