# Supplementary material for: Beyond BMI: central obesity identifies overlooked fatty liver disease risk in adults with normal body mass index undergoing routine health examinations
Source: Front Public Health. 2026 Jun 25;14:1852073. doi: 10.3389/fpubh.2026.1852073 (PMC13346050; doi:10.3389/fpubh.2026.1852073)
Supplement: Supplementary file 1 [file Data_Sheet_1.docx]

Table S1. Baseline characteristics by BMI-waist phenotype.

| **Characteristic** | **Normal BMI / No central obesity  N = 959^1^** | **Normal BMI / Central obesity  N = 263^1^** | **Overweight-obesity / No central obesity  N = 398^1^** | **Overweight-obesity / Central obesity  N = 716^1^** |
| --- | --- | --- | --- | --- |
| **Age, years** | 43.43 ± 11.95 | 48.83 ± 11.45 | 45.65 ± 11.72 | 48.42 ± 11.61 |
| **Sex** |  |  |  |  |
| Female | 527 (55%) | 107 (41%) | 169 (42%) | 236 (33%) |
| Male | 432 (45%) | 156 (59%) | 229 (58%) | 480 (67%) |
| **Smoking status** |  |  |  |  |
| Current | 129 (13%) | 40 (15%) | 58 (15%) | 140 (20%) |
| Former | 145 (15%) | 60 (23%) | 75 (19%) | 139 (19%) |
| Never | 685 (71%) | 163 (62%) | 265 (67%) | 437 (61%) |
| **Drinking status** |  |  |  |  |
| No | 547 (57%) | 127 (48%) | 209 (53%) | 369 (52%) |
| Occasional | 298 (31%) | 97 (37%) | 134 (34%) | 251 (35%) |
| Regular | 114 (12%) | 39 (15%) | 55 (14%) | 96 (13%) |
| **Physical activity** |  |  |  |  |
| High | 185 (19%) | 59 (22%) | 88 (22%) | 147 (21%) |
| Low | 234 (24%) | 66 (25%) | 99 (25%) | 196 (27%) |
| Moderate | 540 (56%) | 138 (52%) | 211 (53%) | 373 (52%) |
| **Body mass index, kg/m^2** | 21.26 ± 1.88 | 22.30 ± 1.23 | 25.66 ± 1.37 | 26.97 ± 2.03 |
| **Waist circumference, cm** | 76.52 ± 7.39 | 92.71 ± 4.92 | 81.52 ± 5.40 | 96.40 ± 6.72 |
| **Systolic blood pressure, mmHg** | 116.97 ± 12.32 | 122.93 ± 12.09 | 123.41 ± 12.46 | 128.66 ± 12.68 |
| **Diastolic blood pressure, mmHg** | 68.75 ± 7.92 | 72.10 ± 8.38 | 73.24 ± 7.78 | 75.95 ± 8.35 |
| **Fasting plasma glucose, mmol/L** | 4.71 ± 0.49 | 5.06 ± 0.46 | 5.14 ± 0.47 | 5.42 ± 0.50 |
| **HbA1c, %** | 5.01 ± 0.28 | 5.19 ± 0.27 | 5.24 ± 0.27 | 5.37 ± 0.29 |
| **Triglycerides, mmol/L** | 1.27 ± 0.49 | 1.67 ± 0.66 | 1.61 ± 0.59 | 2.07 ± 0.83 |
| **HDL-C, mmol/L** | 1.45 ± 0.18 | 1.32 ± 0.17 | 1.33 ± 0.18 | 1.22 ± 0.18 |
| **ALT, U/L** | 21.41 ± 9.46 | 27.77 ± 10.43 | 27.54 ± 11.18 | 35.11 ± 12.58 |
| **Serum uric acid, umol/L** | 313.24 ± 52.80 | 339.73 ± 52.51 | 334.44 ± 54.04 | 365.37 ± 53.56 |
| **Ultrasound steatosis grade** |  |  |  |  |
| None | 852 (89%) | 190 (72%) | 287 (72%) | 347 (48%) |
| Mild | 97 (10%) | 60 (23%) | 83 (21%) | 223 (31%) |
| Moderate-to-severe | 10 (1.0%) | 13 (4.9%) | 28 (7.0%) | 146 (20%) |

Note: Continuous variables are presented as mean ± standard deviation, and categorical variables as n (%). Chinese BMI categories were applied. Central obesity was defined as waist circumference ≥90 cm in men and ≥85 cm in women. Abbreviations: BMI, body mass index; mmHg, millimeters of mercury; HbA1c, hemoglobin A1c; HDL-C, high-density lipoprotein cholesterol; ALT, alanine aminotransferase.

Table S2. Comparison of the primary and sensitivity analyses for the association between central adiposity and fatty liver disease in normal-BMI adults.

| **Analysis** | **Exposure definition** | **Population** | **Adjusted OR (95% CI)** |
| --- | --- | --- | --- |
| Primary analysis | Waist circumference-defined central obesity (vs no central obesity) | Normal-BMI adults | 2.48 (1.73, 3.52) |
| Sensitivity analysis | Waist-to-height ratio ≥0.50 (vs <0.50) | Normal-BMI adults | 2.65 (1.88, 3.78) |
| Note: Values are adjusted odds ratios (95% confidence intervals) estimated using binary logistic regression models among participants with BMI <24 kg/m² according to Chinese adult criteria. Both models were adjusted for age, sex, smoking status, drinking status, and physical activity. In the primary analysis, central obesity was defined as waist circumference ≥90 cm in men and ≥85 cm in women. In the sensitivity analysis, central adiposity was defined as waist-to-height ratio ≥0.50. Fatty liver disease was defined according to abdominal ultrasonography findings. Abbreviations: BMI, body mass index; OR, odds ratio; CI, confidence interval; WHtR, waist-to-height ratio. | | | |

Table S3. Additional analysis separating overweight and obesity in BMI-waist phenotypes

| Variable | Model 1 | Model 2 | Model 3 |
| --- | --- | --- | --- |
| **Panel A. Ultrasound-diagnosed fatty liver disease** | | | |
| Normal BMI / Central obesity | 3.06 (2.18, 4.28) | 2.43 (1.71, 3.43) | 2.46 (1.73, 3.48) |
| Overweight / No central obesity | 2.69 (1.97, 3.67) | 2.39 (1.74, 3.28) | 2.40 (1.74, 3.30) |
| Overweight / Central obesity | 6.75 (5.19, 8.83) | 5.44 (4.14, 7.17) | 5.45 (4.15, 7.19) |
| Obesity / No central obesity | 11.94 (5.65, 26.11) | 9.68 (4.47, 21.71) | 10.16 (4.67, 22.82) |
| Obesity / Central obesity | 15.14 (10.71, 21.61) | 11.77 (8.23, 16.98) | 12.00 (8.37, 17.36) |
| **Panel B. Moderate-to-severe steatosis** | | | |
| Normal BMI / Central obesity | 4.93 (2.15, 11.69) | 3.74 (1.62, 8.93) | 3.74 (1.61, 8.92) |
| Overweight / No central obesity | 5.74 (2.74, 12.85) | 4.95 (2.35, 11.11) | 4.95 (2.35, 11.13) |
| Overweight / Central obesity | 16.36 (8.78, 33.99) | 12.33 (6.57, 25.74) | 12.36 (6.59, 25.82) |
| Obesity / No central obesity | 28.88 (9.74, 82.25) | 21.82 (7.22, 63.35) | 22.76 (7.50, 66.45) |
| Obesity / Central obesity | 49.91 (26.29, 105.16) | 36.23 (18.91, 76.83) | 36.96 (19.25, 78.50) |
| Note: Values are odds ratios (95% confidence intervals) estimated using binary logistic regression. This supplementary analysis separated overweight and obesity according to Chinese adult BMI criteria. The reference group was Normal BMI without central obesity. Overweight was defined as BMI 24.0 to <28.0 kg/m², and obesity was defined as BMI ≥28.0 kg/m². Central obesity was defined as waist circumference ≥90 cm in men and ≥85 cm in women. Model 1 was unadjusted. Model 2 was adjusted for age and sex. Model 3 was additionally adjusted for smoking status, drinking status, and physical activity.  Abbreviations: BMI, body mass index; OR, odds ratio; CI, confidence interval. | | | |

Table S4. Sensitivity analysis of BMI-waist phenotypes and ultrasound-diagnosed FLD after excluding participants who reported regular drinking.

| **Variable** | **Model 1** | **Model 2** | **Model 3** |
| --- | --- | --- | --- |
| **Panel A. Ultrasound-diagnosed FLD** |  |  |  |
| Normal BMI / Central obesity | 2.76 (1.90, 3.97) | 2.20 (1.50, 3.21) | 2.23 (1.52, 3.26) |
| Overweight-obesity / No central obesity | 2.85 (2.07, 3.93) | 2.49 (1.79, 3.47) | 2.51 (1.80, 3.50) |
| Overweight-obesity / Central obesity | 8.75 (6.74, 11.46) | 6.96 (5.32, 9.19) | 7.04 (5.37, 9.29) |
| **Panel B. Moderate-to-severe steatosis** |  |  |  |
| Normal BMI / Central obesity | 4.34 (1.73, 11.06) | 3.32 (1.31, 8.52) | 3.33 (1.31, 8.54) |
| Overweight-obesity / No central obesity | 5.45 (2.50, 12.76) | 4.60 (2.11, 10.82) | 4.63 (2.11, 10.89) |
| Overweight-obesity / Central obesity | 25.13 (13.42, 53.61) | 18.65 (9.89, 39.96) | 19.09 (10.11, 40.95) |

Note: Values are odds ratios (95% confidence intervals) estimated using binary logistic regression after excluding participants who reported regular drinking. A total of 2,032 participants remained in this sensitivity analysis, including 570 participants with ultrasound-diagnosed FLD and 170 participants with moderate-to-severe steatosis. The reference group was normal BMI/no central obesity. Normal BMI was operationalized as BMI <24.0 kg/m² according to Chinese adult criteria. Model 1 was unadjusted. Model 2 was adjusted for age and sex. Model 3 was additionally adjusted for smoking status and physical activity. Drinking status was not included in this sensitivity model because participants reporting regular drinking were excluded and the analysis was restricted to participants reporting no or occasional drinking. This analysis reduced the contribution of participants in the highest available drinking-status category but did not fully exclude alcohol-related liver disease because quantitative alcohol intake was unavailable. Abbreviations: BMI, body mass index; FLD, fatty liver disease; OR, odds ratio; CI, confidence interval.

Table S5. Sensitivity analysis further adjusting for hypertension and dysglycemia.

| **Variable** | **OR (95% CI)** | **P value** |
| --- | --- | --- |
| **Panel A. Ultrasound-diagnosed FLD** |  |  |
| Normal BMI / Central obesity | 2.36 (1.66, 3.35) | <0.001 |
| Overweight-obesity / No central obesity | 2.42 (1.77, 3.31) | <0.001 |
| Overweight-obesity / Central obesity | 5.39 (4.12, 7.10) | <0.001 |
| **Panel B. Moderate-to-severe steatosis** |  |  |
| Normal BMI / Central obesity | 3.48 (1.49, 8.32) | 0.004 |
| Overweight-obesity / No central obesity | 5.15 (2.52, 11.35) | <0.001 |
| Overweight-obesity / Central obesity | 13.34 (7.14, 27.79) | <0.001 |

Note: Values are odds ratios (95% confidence intervals) estimated using binary logistic regression. This sensitivity analysis further adjusted Model 3 for hypertension and dysglycemia to account for broader metabolic status. The model was adjusted for age, sex, smoking status, drinking status, physical activity, hypertension, and dysglycemia. The reference group was Normal BMI / No central obesity. Hypertension was defined as systolic blood pressure ≥130 mmHg, diastolic blood pressure ≥85 mmHg, self-reported history of hypertension, or current use of antihypertensive medication. Dysglycemia was defined as fasting plasma glucose ≥5.6 mmol/L, hemoglobin A1c ≥5.7%, self-reported history of diabetes, or current use of glucose-lowering medication. Abbreviations: BMI, body mass index; FLD, fatty liver disease; OR, odds ratio; CI, confidence interval.

**
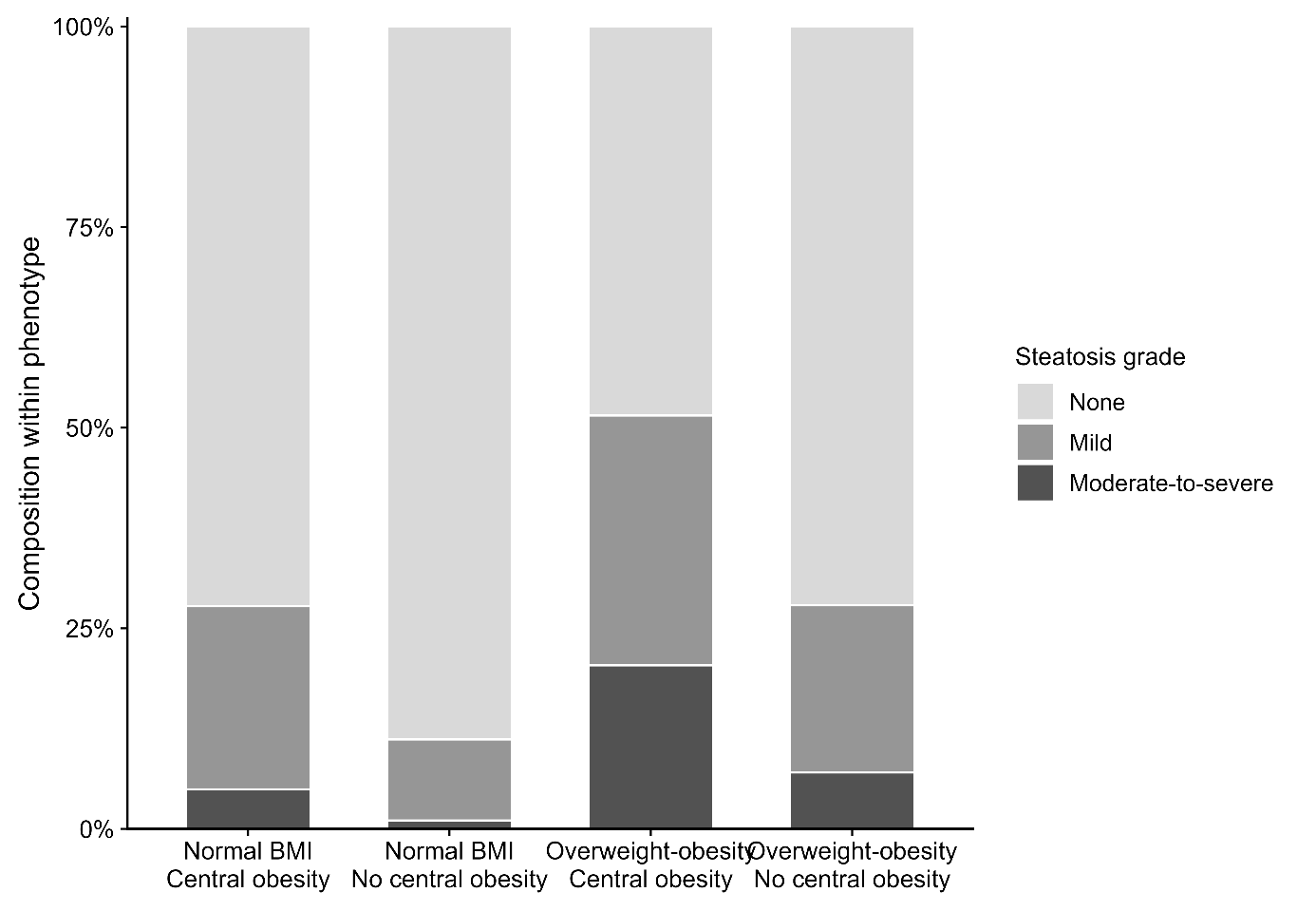
**

**Figure S1. Distribution of ultrasound steatosis grades across BMI-waist phenotypes.**

Stacked bars show the within-phenotype distribution of ultrasound steatosis grades (none, mild, and moderate-to-severe) across BMI-waist phenotype groups. Proportions within each phenotype sum to 100%. BMI categories were defined according to Chinese adult criteria. Central obesity was defined as waist circumference ≥90 cm in men and ≥85 cm in women.
